# The Role of Glucagon-Like Peptide-1 Receptor Agonists in the Treatment of Cardiovascular-Kidney-Metabolic Syndrome

**DOI:** 10.1016/j.jacadv.2025.102465

**Published:** 2026-01-28

**Authors:** Sonal Kumar, John E. Anderson, Andrea Coviello, Francisco Lopez-Jimenez, George L. Bakris

**Affiliations:** a Weill Cornell Medical College, Cornell University, New York, New York, USA; b The Frist Clinic, Nashville, Tennessee, USA; c University of North Carolina, Chapel Hill, North Carolina, USA; d Mayo Clinic, Rochester, Minnesota, USA; e The University of Chicago, Chicago, Illinois, USA

**Keywords:** cardiovascular disease, cardiovascular-kidney-metabolic syndrome, chronic kidney disease, diabetes, liver dysfunction, obesity

## Abstract

It has long been acknowledged that the risk of cardiovascular disease is impacted by a complex interplay between the heart and kidneys. The relationship among cardiovascular disease, chronic kidney disease, and metabolic diseases, including obesity and diabetes, has now been recognized as “cardiovascular-kidney-metabolic (CKM) syndrome”. Specialist clinicians have historically approached these disorders as separate diseases, with individual treatment plans specific to dysfunction of each organ system. However, the recognition of a syndrome of CKM dysfunction and the advent of therapeutic agents with pleiomorphic effects across multiple organ systems, such as glucagon-like peptide-1 receptor agonists, which target various components of CKM, may encourage clinicians to take a more holistic approach to treatment of people with CKM.

## Highlights

• Traditionally, cardiometabolic disorders and other associated metabolic diseases have been treated as individual disease components.
• The cardiometabolic association among cardiovascular disease, chronic kidney disease, and other metabolic disorders has been recently described as CKM syndrome.
• Raising awareness to clinicians of the benefits of therapeutic drug classes with multiple beneficial effects on CKM syndrome may encourage a holistic approach to treatment.

The appreciation of the heart/kidney interaction and the interplay of these organs on cardiovascular (CV) risk have been acknowledged for over 2 decades.^1^^,^^2^ In more recent years, this important inter-relationship between cardiovascular disease (CVD), chronic kidney disease (CKD), and metabolic disorders such as obesity and diabetes has become increasingly recognized.^3^ In 2023, the American Heart Association described this cardiometabolic association as cardiovascular-kidney-metabolic (CKM) syndrome.^3^

**Figure 1:**
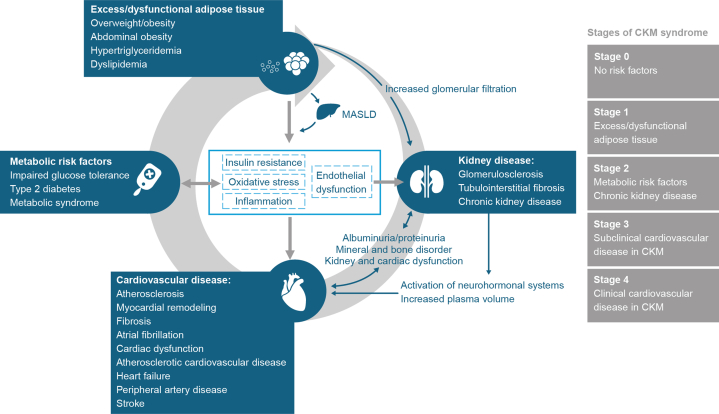
**The Interplay of Cardiovascular-Kidney-Metabolic Syndrome**^3^^,^^4^ CKM = cardiovascular-kidney-metabolic; MASLD = metabolic dysfunction-associated steatotic liver disease.

CKM has been defined (Figure 1)^4^ as a systemic disorder characterized by pathophysiological interactions among metabolic disorders, CKD, and the CV system leading to multi-organ dysfunction and a high rate of adverse CV outcomes.^3^ CKM has 4 recognized stages of progression: stage 0, no risk factors; stage 1, excess/dysfunctional adipose tissue; stage 2, metabolic risk factors and CKD; stage 3, subclinical CVD in CKM syndrome; and stage 4, clinical CVD in CKM syndrome.^3^ Historically, CV and metabolic disorders have been treated according to condition-specific recommendations, often under the guidance of different health care professionals.^5^ In 2022 and 2024, an international taskforce including specialists in cardiology, nephrology, endocrinology, and primary care physicians collaborated across specialties to publish the DCRM (diabetes, cardiorenal, and/or metabolic) Multispecialty Practice Recommendations, a document that would provide a set of evidence-based integrated recommendations for specialists treating patients within the spectrum of cardiorenal and metabolic diseases.^5^^,^^6^ In recent years, medications such as glucagon-like peptide-1 receptor agonists (GLP-1 RAs), originally developed for the treatment of type 2 diabetes (T2D), have been shown in large-scale outcome trials to benefit various markers of CV risk beyond hyperglycemia, such as elevated blood pressure and excess body weight.^7^, ^8^, ^9^ Furthermore, GLP-1 RAs developed and approved at higher doses for the treatment of obesity have demonstrated beneficial effects on CV risk profile and outcomes beyond weight loss.^10^

The increased recognition of the inter-related nature of metabolic disorders has led to further research, hence the concept of multimorbidity CKM syndrome.^11^, ^12^, ^13^ The recognition of the CKM syndrome and poor CKM health as potential contributors to a health care and financial burden,^3^ in addition to an increasing body of data on treatments available for various inter-related cardiometabolic diseases, may now enable specialists to take a more holistic approach to the management of global CKM risk. This review will explore the concept of multimorbid disease and assess the role of various GLP-1 RAs as a treatment option within the CKM syndrome context (Central Illustration). As this is a review article, which uses data from previously published manuscripts only, there was no need for ethical (Institutional Review Board) approval.

**Figure 2:**
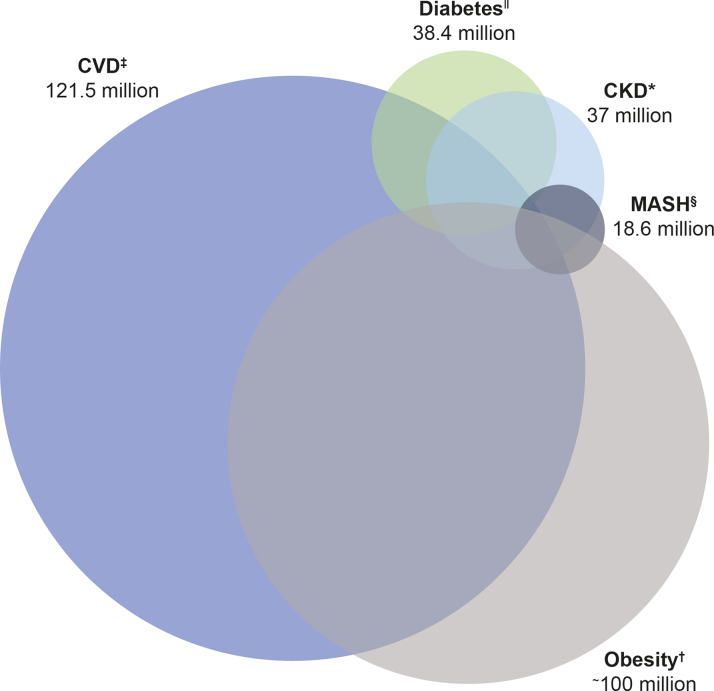
**Conceptualization of the Prevalence of Key CKM Multimorbidities Within the Total U.S. Population**^14^, ^15^, ^16^, ^17^, ^18^**(Data From 2015 to 2023)** References: ∗14; ^†^15; ^‡^16 (excluding hypertension, includes CHD, HF, and stroke only); ^§^17; ^ǁ^18. Please note: this figure is a conceptual figure for illustrative purposes and is not drawn to scale. CHD = coronary heart disease; CKD = chronic kidney disease; CVD = cardiovascular disease; HF = heart failure; MASH = metabolic dysfunction-associated steatohepatitis; other abbreviation as in Figure 1.

**Central Illustration:**
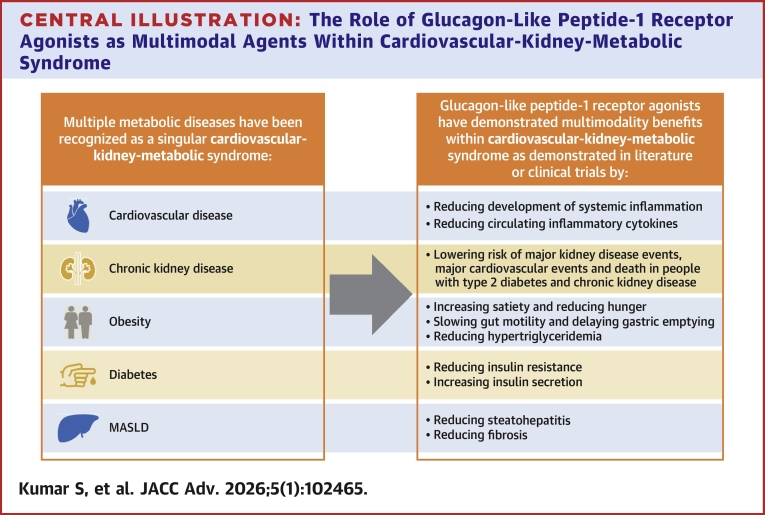
**The Role of Glucagon-Like Peptide-1 Receptor Agonists as Multimodal Agents Within Cardiovascular-Kidney-Metabolic Syndrome** Abbreviation as in Figure 1.

## The prevalence of CKM multimorbidities

Globally, CVD is a leading cause of death in both men and women. In 2019, approximately 17.9 million people died due to CVD, which accounted for 32% of all global deaths.^19^ Furthermore, in 2021, coronary artery disease was the leading cause in 20% of all deaths in adults under 65 years of age in the United States.^20^ CVD, CKD, and metabolic diseases (such as obesity, diabetes, and metabolic dysfunction-associated steatohepatitis [MASH]) are highly prevalent in the United States (Figure 2).^14^, ^15^, ^16^, ^17^, ^18^, ^19^, ^20^ The prevalence of obesity and T2D has increased in recent decades; indeed, both are recognized as 2 of the main metabolic disorders within the CKM syndrome.^3^^,^^15^^,^^20^ Recent research suggested that in the period of 2017-2018, 34.3 million adults in the United States had ≥2 metabolic diseases.^13^ This analysis did not include obesity or metabolic dysfunction-associated steatotic liver disease (MASLD), suggesting the true prevalence of these conditions is likely to be higher still. People with T2D have high rates of atherosclerotic cardiovascular disease (ASCVD), CKD, and heart failure (HF).^11^^,^^12^^,^^21^

Recognized as a risk factor for CVD, T2D increases the risk of developing any CVD by up to 4 times compared to people without T2D.^22^ It has been reported that 14.9% of people with T2D have HF, 21.2% have coronary heart disease, and 7.6% have stroke.^21^ People at risk of CKM syndrome include those at risk of CVD with metabolic risk factors, with or without CKD, and those with existing CKD related to other metabolic risk factors,^4^ and it is estimated that 33 to 40% of adults in the United States are affected by CKM syndrome.^23^^,^^24^ With a recognized interconnection among the different cardiorenal/metabolic diseases of the heart, liver, kidney, and associated risk factors of obesity and diabetes, a shared pathophysiology has been suggested.^3^

## CKM multimorbidities:a shared pathophysiology

There is an increasing understanding that the various manifestations of disease within CKM syndrome involve inter-related pathophysiological processes, often acting in “vicious cycles.”^25^

Obesity is associated with other chronic diseases, including metabolic and hepatic dysfunction, as well as CVD.^26^ Thus, by this association, obesity represents an important therapeutic target for multimorbidity prevention.^25^^,^^27^^,^^28^ Insulin resistance has been associated with obesity, but the precise mechanisms that lead to reduced insulin sensitivity in dysfunctional adipose tissue are not fully understood and continues to be a topic of much research.^29^ For instance, changes in adipose tissue fatty acid and adipokine metabolism contribute to the development of insulin resistance, which is believed to contribute to increased blood pressure via vasoconstriction via an increase in circulating free fatty acids.^3^^,^^29^^,^^30^ Additionally, inflammation due to cytokine production by dysregulated adipose tissue (eg, tumor necrosis factor-alpha, interleukin-6, interleukin-1ß) may further contribute to insulin resistance.^29^^,^^30^ When multiple morbidities manifest, the risk of adverse outcomes is greatly increased.^31^^,^^32^ A pooled analysis of 16 cohort studies within the United States and Europe found that the risk of cardiometabolic multimorbidity (developing ≥2 conditions: T2D, coronary heart disease, and stroke) in people with overweight (body mass index [BMI] 25 to 29.9 kg/m^2^) was double that of people with a healthier weight (BMI: 20-24.9 kg/m^2^) (OR: 2.0; 95% CI: 1.7-2.4), 5 times higher for people with class I obesity (BMI: 30-34.9 kg/m^2^) (OR: 4.5; 95% CI: 3.5-5.8), and 15 times higher for people with class II and III obesity combined (BMI ≥35 kg/m^2^) (OR: 14.5; 95% CI: 10.1-21.0); *P* < 0.0001 for all.^27^ Although understanding of the underlying shared pathophysiological pathways that connect obesity and CKD is limited, obesity is believed to contribute to the inflammation, hemodynamic alterations, and metabolic dysregulation that lead to a decline in renal function.

CKD is defined as abnormalities of kidney structure or function, present for a minimum of 3 months, with implications for health including one or more of the following: albuminuria (albumin-to-creatinine ratio ≥30 mg/g [≥3 mg/mmol]); urine sediment abnormalities, persistent hematuria, electrolyte, and other abnormalities due to tubular disorders; abnormalities detected by histology; structural abnormalities detected by imaging, history of kidney transplantation, and glomerular filtration rate <60 mL/min/1.73 m^2^.^33^ CKD is a well-known CV risk factor and should be managed to slow progression with the goal of reducing CV risk and preserving renal function.^34^^,^^35^ The proposed mechanisms by which CKD leads to CVD are complex. Recent theories include vascular senescence and senescence of peripheral blood cells, which leads to early-stage vascular pathology such as vascular calcification in people with CKD.^36^^,^^37^ However, 2 major mechanisms are believed to contribute to the development of CVD in CKD.^38^ Firstly, peptide post-translation modification, calcification, dyslipidemia, coagulation, toxic metabolites, endothelial dysfunction, and inflammatory cytokines released in response to kidney injury or insufficiency all may contribute to CV changes.^36^^,^^38^ Secondly, oxidative stress and CKD-associated mediators such as uremic toxins may lead to CV damage (valve disease and myocardial fibrosis).^38^ In combination with genetic factors and age, coexisting T2D and CKD all contribute to progressive CVD, leading to greater morbidity and mortality.^38^^,^^39^

CVD remains the leading cause of mortality in people with MASLD and MASH.^40^, ^41^, ^42^ Proposed shared pathophysiological mechanisms between CVD and MASLD include endothelial dysfunction, oxidative stress, lipid metabolism dysfunction, increased hepatic insulin resistance, and increased systemic inflammation.^43^ An increase in circulating free fatty acids, low-density lipoproteins, and very low-density lipoproteins due to altered lipid metabolism, in conjunction with oxidative stress and increased lipogenesis, leads to increased CVD risk in people with MASH.^40^

Considering the various, complex shared pathophysiologies between various CKM multimorbidities, physicians should consider a holistic approach when treating affected people.^1^^,^^34^ Multimodality therapeutic agents, such as GLP-1 RAs, with various indications, may offer a beneficial treatment option for people with CKM syndrome.

## GLP-1 RAs: the discovery of benefits beyond reduction of hyperglycemia

Glucagon-like peptide-1 (GLP-1) is an endogenous peptide hormone secreted in response to nutrient ingestion, which stimulates glucose-dependent insulin release in the pancreas.^44^^,^^45^ GLP-1 also induces satiety, regulating appetite centrally via the afferent vagal nerve system, inhibits postprandial glucagon secretion from α-cells in the pancreas, and are associated with delayed gastric emptying.^46^ GLP-1 induces its effects by acting as an agonist at the GLP-1 receptor, located in multiple sites, including in the central nervous system, enteric nervous system, heart, and pancreas.^44^^,^^47^ Endogenous GLP-1 has a very short half-life and is subject to degradation by the enzyme dipeptidyl peptidase-4.^48^ The first GLP-1 RA to be developed commercially was the synthetic short-acting GLP-1 RA exenatide (a form of the peptide exendin-4),^49^ approved by the U.S. Food and Drug Administration (FDA) in 2005 for people with T2D.^50^ Treatment with exenatide was associated with a significant and sustained dose-dependent reduction in body weight.^51^ The human analog GLP-1 RAs, including liraglutide, dulaglutide, and semaglutide, are longer acting, therefore extending the beneficial pharmacologic effects on metabolism, appetite, and weight regulation.^48^^,^^52^ While GLP-1 RAs were first approved to treat T2D, clinical trials have shown that GLP-1 RAs are effective in achieving clinically relevant weight loss, leading to the development of some GLP-1 RAs for the treatment of overweight and obesity, with FDA approval for use in chronic weight management in patients with obesity or overweight with at least one weight-related condition separate from T2D.^53^^,^^54^ The glucose-dependent action of GLP-1 RAs in the pancreas minimizes the risk of hypoglycemia in people without T2D.^55^

The mechanism of action by which GLP-1 RAs exert effects within the CV system is under continued investigation, but it is believed to be multifactorial and driven by multiple interrelated pathways. GLP-1 RAs have shown anti-inflammatory effects in the progression of atherosclerosis as shown in animal studies; both liraglutide and semaglutide exposure demonstrated a significant reduction in the development of plaque lesion, and semaglutide decreased plasma levels of circulating inflammatory cytokines (tumor necrosis factor-alpha, interferon-γ).^56^ Additionally, GLP-1 RAs improve blood flow, increase vasodilation to improve endothelial function, alongside reductions in apoptosis, hypertension, and hypertriglyceridemia. Mechanisms by which GLP-1RAs induce these actions are by increased vascular nitric oxide, increased diuresis and natriuresis, and via decreased glucagon levels.^57^^,^^58^

The renal-protective effects of GLP-1 RAs, through both direct and indirect mechanisms of action, have been hypothesized.^59^ Antihyperglycemic effects in conjunction with an impact on weight, blood pressure, and lipid metabolism are all believed to contribute to the indirect nephroprotective properties.^59^ Direct mechanisms underlying the renal-protective effects of GLP-1 RAs include attenuation of oxidative stress and inflammation, natriuresis, and a decrease in intraglomerular pressure.^59^

Knowledge of the mechanism of action by which GLP-1 RAs exert influence on MASLD is still limited; however, they have been shown to reduce steatosis by reducing the accrual of triglycerides in the liver, lessen inflammation by down-regulating the levels of circulating inflammatory cytokines, and slow the progression of fibrosis by reducing hepatocyte cell death, decreasing plasma fibroblast growth hormones and inhibition of cell apoptosis.^60^^,^^61^

The established CV outcome benefits (such as reduction in major adverse cardiovascular events [MACE]) achieved by GLP-1 RAs^62^, ^63^, ^64^ have resulted in several pharmacological studies and hypotheses regarding the impact of GLP-1 RAs on ASCVD and the manifestations of disease within the CKM syndrome.^65^, ^66^, ^67^ These include weight and blood pressure reduction,^68^ albuminuria reduction,^69^ anti-inflammatory effects,^70^ and beneficial impacts on postprandial dysmetabolism.^71^

The recognition of these effects on all the major organ systems impacted by cardiometabolic diseases within the CKM syndrome and the number of trials currently exploring the effects of GLP-1 RAs suggest that GLP-1 RAs may become an important treatment option for many facets of the CKM syndrome.

## Trial data supporting GLP-1 RAs in the treatment of CKM multimorbidity components

### MACE outcomes

In 2008, following concerns over the CV safety of rosiglitazone, an antidiabetic drug within the thiazolidinedione class, the FDA mandated placebo-controlled CV outcome trials (CVOTs) for all new T2D medications, including GLP-1 RAs.^72^ Several large CVOTs (Table 1),^8^^,^^10^^,^^64^^,^^73^, ^74^, ^75^, ^76^, ^77^, ^78^, ^79^ meta-analyses of CVOTs, and key randomized controlled trials (RCTs) with varying inclusion criteria, demographics, and endpoints have reported results on treatment with GLP-1 RA or GLP-1/glucose-dependent insulinotropic polypeptide agonist for the related metabolic disease states; various CVOTs have demonstrated that several GLP-1 RA analogs reduce MACE and other endpoints.^9^^,^^91^^,^^92^ The beneficial effects of semaglutide, independent of diabetes, among people with CVD and overweight or obesity^93^ without diabetes have been demonstrated in a recent RCT.^10^ The SELECT (Semaglutide Effects on Cardiovascular Outcomes in People With Overweight or Obesity) trial recently reported a reduction in MACE of 20% with subcutaneous once-weekly semaglutide 2.4 mg compared with placebo.^94^ A reduced incidence of composite MACE (including CV death, nonfatal myocardial infarction, or nonfatal stroke) was observed in people treated with semaglutide 2.4 mg vs placebo (6.5% vs 8.0% [HR: 0.80; 95% CI: 0.72-90; *P* < 0.001]).^10^ A meta-analysis of 8 CVOTs reported an overall reduction in MACE (HR: 0.86; 95% CI: 0.79-0.94; *P* = 0.006) with treatment with GLP-1 RAs in people with T2D.^95^

**Table 1:** Summary of Data From Key Completed Outcome Trials Investigating GLP-1 RAs in Diseases Within CKM Syndrome

Trial Name (Publication Year)	Population	Treatment Arms	Main Efficacy Findings in Those Treated With GLP-1 RA vs Placebo
Cardiovascular outcome trials in people with T2D			
AMPLITUDE-O^73^ (2021)	T2D, history of CVD^a^ or current kidney disease (eGFR 25-59.9 mL/min/1.73 m^2^ of body surface area), ≥1 additional CV risk factor	Once-weekly subcutaneous efpeglenatide 4 mg or 6 mg vs placebo	*Incidence of MACE (first occurrence of death from CV or unknown causes, nonfatal MI, or nonfatal stroke) was lower in the efpeglenatide vs placebo group* *7.0% vs 9.2% (HR: 0.73; 95% CI: 0.58-0.92)* *P < 0.001 for noninferiority; P = 0.007 for superiority*
ELIXA^74^ (2015)	T2D, recent acute coronary syndrome^b^	Once-daily subcutaneous lixisenatide 10 μg for 2 weeks then increased to 20 μg vs placebo	Incidence of MACE (first occurrence of CV death, nonfatal MI, nonfatal stroke, or hospitalization for unstable angina) did not differ significantly between groups 13.4% vs 13.2% (HR: 1.02; 95% CI: 0.89-1.17) *P < 0.001 for noninferiority; P = 0.81 for superiority*
EXSCEL^75^ (2017)	T2D, with or without previous CVD^c^	Once-weekly subcutaneous exenatide 2 mg vs placebo	Incidence of MACE (first occurrence of CV death, nonfatal MI, or nonfatal stroke) did not differ significantly between groups 11.4% vs 12.2% (HR: 0.91; 95% CI: 0.83-1.00) *P < 0.001 for noninferiority; P = 0.06 for superiority*
Harmony Outcomes^76^ (2018)	T2D and established CVD^d^	Once-weekly subcutaneous albiglutide 30–50 mg vs placebo in addition to standard of care	*Incidence of MACE (first occurrence of CV death, nonfatal MI, or nonfatal stroke) was significantly lower in the albiglutide vs placebo group* *7% vs 9% (HR: 0.78; 95% CI: 0.68-0.90)* *P < 0.001 for noninferiority; P = 0.006 for superiority*
LEADER^8^ (2016)	T2D, high CV risk^e^	Once-daily subcutaneous liraglutide 1.8 mg vs placebo	*Incidence of MACE (first occurrence of CV death, nonfatal MI, or nonfatal stroke) was significantly lower in the liraglutide vs placebo group* *13.0% vs 14.9% (HR: 0.87; 95% CI: 0.78-0.97)* *P < 0.001 for noninferiority; P = 0.01 for superiority*
PIONEER 6^77^ (2019)	T2D, high CV risk^f^	Once-daily oral semaglutide 14 mg vs placebo	*Confirmed noninferiority of semaglutide vs placebo for MACE (first occurrence of CV death, nonfatal MI, or nonfatal stroke)* *3.8% vs 4.8% (HR: 0.79; 95% CI: 0.57-1.11)* *P < 0.001 for noninferiority; P = 0.17 for superiority*
REWIND^78^ (2019)	T2D, previous CVD or CV risk factors^g^	Once-weekly subcutaneous dulaglutide 1.5 mg vs placebo	*Incidence of MACE (first occurrence of death from CV or unknown causes, nonfatal MI, or nonfatal stroke) was significantly lower in the dulaglutide vs placebo group* *12.0% vs 13.4% (HR: 0.88; 95% CI: 0.79-0.99; P = 0.026)*
SUSTAIN 6^64^^,^^79^ (2016)	T2D, ≥50 years: established CVD^h^ or stage 3+ CKD, ≥60 years: ≥1 CV risk factor as determined by the investigator	Once-weekly subcutaneous semaglutide 0.5–1.0 mgvs placebo	*Incidence of MACE (first occurrence of CV death, nonfatal MI, or nonfatal stroke) was significantly lower in the semaglutide group vs placebo group* *6.6% vs 8.9% (HR: 0.74; 95% CI: 0.58-0.95)* *P < 0.001 for noninferiority; P = 0.02 for superiority*
SOUL^80^^,^^81^	People aged ≥50 years with T2D, HbA_1c_ 6.5–10%; established vascular disease^i^ and CKD^j^	Oral daily semaglutide 14 mg vs placebo	*Incidence of MACE (first occurrence of CV death, nonfatal MI, or nonfatal stroke) was significantly lower in the semaglutide group vs placebo group* *12.0% vs 13.8% (HR: 0.86; 95% CI: 0.77-0.96)* *P = 0.006 for superiority*
SURPASS-CVOT^82^^,^^83^^,^^167^	People aged ≥40 years with T2D and established ASCVD	Once-weekly tirzepatide 15 mg vs dulaglutide 1.5 mg	*Incidence of MACE (first occurrence of CV death, MI, or stroke) was not significantly different between the groups, demonstrating noninferiority of tirzepatide vs dulaglutide* *HR: 0.92; 95.3% CI: 0.83–1.01; P = 0.086*
Cardiovascular outcome trials in people with obesity without T2D			
SELECT^10^ (2023)	Overweight or obesity (BMI ≥27 kg/m^2^), established CVD^k^, no prior diabetes history	Once-weekly subcutaneous semaglutide 2.4 mg vs placebo	*Incidence of MACE (first occurrence of CV death, nonfatal MI, or nonfatal stroke) was significantly lower in the semaglutide vs placebo group* *6.5% vs 8.0% (HR: 0.80; 95% CI: 0.72-0.90; P < 0.001)*
Outcomes in people with obesity and HFpEF			
SUMMIT^84^, ^85^, ^86^	People aged ≥40 years with stable HF and LVEF ≥50%, elevated NT-proBNP, eGFR <70 mL/min/1.73 m^2^ at screening, or HF decompensation within 12 months of screening, stable dose of HF medications within 4 weeks of screening, BMI ≥30 kg/m^2^, 6MWD 100–425 m, KCCQ-CSS ≤80, and obesity with or without T2D	Subcutaneous tirzepatide 5 mg, 10 mg, or 15 mg vs placebo	*The relative risk reduction of time to first occurrence of heart failure outcomes was lower with tirzepatide vs placebo* *Relative risk reduction: 38% (HR: 0.62; 95% CI: 0.41-0.95; P = 0.026)*
Renal outcome trials in people with T2D and CKD			
FLOW^87^ (2024)	T2D and CKD^l^	Once-weekly subcutaneous semaglutide 1.0 mg vs placebo	*Risk of a major kidney disease event was lower in the semaglutide group vs placebo group* *331 first events vs 410 first events (HR: 0.76; 95% CI: 0.66-0.88; P = 0.0003)*

**Trial (Phase)**	**Population**	**Treatment Arms**	**Efficacy Outcomes**

Liver outcome trials in people with biopsy-defined MASH and fibrosis stage 2 or 3			
ESSENCE (phase 3, part 1 with n = 800; part 2 ongoing)^88^	Histologically documented steatohepatitis and liver fibrosis stage 2 or 3, according to the NASH CRN classification, and a nonalcoholic fatty liver disease activity score (NAS) of 4 or more	Once-weekly subcutaneous semaglutide 2.4 mg vs placebo in addition to standard care for MASH and related coexisting illnesses	*Resolution of steatohepatitis without worsening of fibrosis was greater in the semaglutide group vs placebo group* *62.9% vs 34.3% (ETD: 28.7% points; 95% CI: 21.1-36.2; P < 0.001)* *Reduction in liver fibrosis without worsening of steatohepatitis was greater in the semaglutide group vs placebo group* *36.8% vs 22.4% (ETD: 14.4% points; 95% CI: 7.5-21.3; P < 0.001)*
SYNERGY-NASH (phase 2, n = 190)^89^	Biopsy-confirmed MASH and stage F2 or F3 (moderate or severe) fibrosis	Once-weekly subcutaneous tirzepatide (5 mg, 10 mg, or 15 mg) vs placebo	*Resolution of MASH without worsening of fibrosis was greater in the tirzepatide group vs placebo* *Tirzepatide 5 mg: 44% vs 10% (ETD: 34% points; 95% CI: 17-50; P < 0.001)* *Tirzepatide 10 mg: 56% vs 10% (ETD: 46% points; 95% CI: 29-62; P < 0.001)* *Tirzepatide 15 mg: 62% vs 10% (ETD: 53% points; 95% CI: 37-69; P < 0.001)*

**Trial Name (Publication Year)**	**Population**	**Treatment Arms**	**Main Efficacy Findings in Those Treated With GLP-1 RA vs Placebo**

Outcomes in people with T2D			
STRIDE et al (2025)^90^^,^^169^	People aged ≥18 years with T2D and symptomatic PAD with intermittent claudication corresponding to Fontaine stage IIa (Rutherford classification grade I, category 1, and 2)^e^	Once-weekly subcutaneous semaglutide 1.0 mg or placebo	*The ratio to baseline of the maximum walking distance on a constant load treadmill test at week 52 was significantly greater in the semaglutide vs placebo group* *1.21 (IQR: 0.95-1.55) vs 1.08 (IQR: 0.86-1.36); ETR: 1.13; 95% CI: 1.061.21; P = 0.0004*

*Italic* “key findings” cells indicate that there was a difference in effect between GLP-1 RA and placebo. For all of the listed trials in Table 1, in both treatment arms, treatment was in addition to CV risk reduction standard of care for all pertinent comorbid conditions (eg, CVD, T2D) according to local clinical practice guidelines at the time of trial conduct.

6MWD = 6-minute walk distance; BMI = body mass index; CKD = chronic kidney disease; CKM = cardiovascular-kidney-metabolic; CV = cardiovascular; CVD = cardiovascular disease; CVOT = cardiovascular outcome trial; eGFR = estimated glomerular filtration rate; ETD = estimated treatment difference; ETR = estimated treatment ratio; GLP-1 RA = glucagon-like peptide-1 receptor agonist; HbA1c = glycated hemoglobin; HF = heart failure; HFpEF = heart failure with preserved ejection fraction; KCCQ-CSS = Kansas City Cardiomyopathy Questionnaire Clinical Summary Score; LVEF = left ventricular ejection fraction; MACE = major adverse cardiovascular events; MASH = metabolic dysfunction-associated steatohepatitis; MI = myocardial infarction; NASH CRN = Nonalcoholic Steatohepatitis Clinical Research Network; NT-proBNP = N-terminal pro B-type natriuretic peptide; PAD = peripheral artery disease; T2D = type 2 diabetes.

a Defined as coronary artery disease, stroke, or PAD.

b People with T2D who experienced a MI or who had been hospitalized for unstable angina within the preceeding 180 days to receive lixisenatide or placebo along with locally determined standards of care.

c Previous CV events were defined as a history of major clinical manifestation of coronary artery disease, ischemic cerebrovascular disease, or atherosclerotic PAD.

d Defined as MI, no < 50% stenosis in one coronary artery or more, or previous coronary revascularization. Participants were also eligible if history of cerebrovascular (ischemic stroke, >50% carotid artery stenosis, or a previous carotid vascular procedure), or peripheral arterial circulation (intermittent claudication and an ankle to brachial index <0.9, nontraumatic amputation, or a previous peripheral vascular procedure) who had a glycated hemoglobin concentration >7.0% (53 mmol/mol).

e As determined by the investigator.

f Defined as age ≥50 years with established CVD or CKD, or ≥60 years with CV risk factors only.

g Defined as people aged ≥50 years with vascular disease, ≥55 years with myocardial ischemia, coronary, carotid, or lower extremity artery stenosis >50%, left ventricular hypertrophy, eGFR <60 mL/min/1.73 m^2^, or albuminuria; and ≥60 years with ≥2 of tobacco use, dyslipidemia, hypertension, or abdominal obesity.

h Defined as previous CV, cerebrovascular, or peripheral vascular disease.

i Defined as coronary artery disease, cerebrovascular disease, or PAD.

j Male ≥50 years or female ≥50 years of age with kidney disease (eGFR: 25.0-59.9 mL/min/1.73 m^2^).

k Defined as previous MI, previous stroke, or symptomatic PAD defined as intermittent claudication with ankle–brachial index <0.85, peripheral arterial revascularization procedure, or amputation due to atherosclerotic disease.

l Defined by an eGFR of 50 to 75 mL/min/1.73 m^2^ and a urinary albumin-to-creatinine ratio (with albumin measured in milligrams and creatinine measured in grams) of >300 and <5,000 or an eGFR of 25 to <50 mL/min/1.73 m^2^ and a urinary albumin-to-creatinine ratio of >100 and <5,000).

### Renal outcomes

Supported by retrospective analyses, GLP-1 RAs have been identified as a potentially effective treatment option for people with diabetic kidney disease based on promising results from previous analyses, such as the SUSTAIN 6 and LEADER (Liraglutide Effect and Action in Diabetes: Evaluation of Cardiovascular Outcome Results) trials.^8^^,^^34^^,^^64^ The multicenter open-label AWARD-7 trial investigated the safety and efficacy of once-weekly subcutaneous dulaglutide (0.75 mg and 1.5 mg) vs insulin glargine, both in combination with insulin lispro, in people with T2D and moderate-to-severe CKD (stages 3-4). At 52 weeks, estimated glomerular filtration rate (eGFR) (calculated by cystatin C equation [mL/min/1.73 m^2^]) was higher with both dulaglutide 1.5 mg and dulaglutide 0.75 mg (least square means 33.8 mL/min/1.73 m^2^ [SE: 0.7]; *P* = 0.005 vs insulin glargine, and 33.8 mL/min/1.73 m^2^ [SE: 0.7]; *P* = 0.009 vs insulin glargine, respectively), than with insulin glargine (31.3 mL/min/1.73 m^2^ [SE: 0.7]).^96^ Furthermore, the effect of treatment with semaglutide on kidney outcomes in people with CKD and T2D was investigated in a global RCT (FLOW [Evaluate Renal Function with Semaglutide Once Weekly] trial) to assess the potential cardiorenal-protective effects vs placebo.^97^ Treatment with semaglutide led to a lower risk of major kidney disease events (composite of the onset of kidney failure [dialysis, transplantation, or eGFR <15 mL/min/1.73 m^2^], ≤50% reduction in the eGFR from baseline, or death from kidney-related or CV causes) (first events: semaglutide: 331 vs placebo: 410 [HR: 0.76; 95% CI: 0.66-0.88; *P* = 0.0003) and a lower risk of first kidney-specific primary outcome component events (HR: 0.79; 95% CI: 0.66-0.94) and death from CV causes (HR: 0.71; 95% CI: 0.56-0.89).^87^

### Liver outcomes

For people with MASH, a phase 2 trial has evaluated the efficacy and safety of subcutaneous liraglutide.^98^ In the phase 2 LEAN (Liraglutide safety and efficacy in patients with non-alcoholic steatohepatitis) trial, 9 (39%) of the 23 people who received liraglutide (1.8 mg daily) and underwent an end-of-treatment liver biopsy had resolution of MASH compared with 2 (9%) of 22 people treated with placebo (relative risk: 4.3 [95% CI: 1.0-17.7], *P* = 0.019).^98^ The ongoing 2-part phase 3 ESSENCE (The Effect of Semaglutide in Subjects With Non-cirrhotic Non-alcoholic Steatohepatitis) trial is investigating the effect of subcutaneous once-weekly semaglutide 2.4 mg vs placebo in patients with MASH (outcomes are time to resolution of steatohepatitis with no worsening of liver fibrosis, and reduction in liver fibrosis and no worsening of steatohepatitis (part 1), and cirrhosis-free survival (part 2).^99^^,^^100^ In part 1, semaglutide improved liver histology compared with placebo after 72 weeks. In people treated with semaglutide (n = 534), there was a greater proportion with resolution of steatohepatitis and no worsening of liver fibrosis (62.9% vs 34.3%; *P* < 0.001), and a greater proportion with a reduction in liver fibrosis and no worsening of steatohepatitis (36.8% vs 22.4%; *P* < 0.001) vs placebo (n = 266), respectively.^88^ The full results from part 2 are expected in 2029.^88^ The efficacy of the dual glucose-dependent insulinotropic polypeptide agonist tirzepatide vs placebo in patients with MASH and moderate or severe fibrosis was investigated in the SYNERGY-NASH (A Randomized, Double-Blind, Placebo-Controlled Phase 2 Study Comparing the Efficacy and Safety of Tirzepatide Versus Placebo in Patients With Nonalcoholic Steatohepatitis) phase 2 trial.^89^ Treatment with once-weekly tirzepatide (5 mg, 10 mg, or 15 mg; n = 142) was more effective regarding resolution of MASH without worsening of fibrosis vs placebo (n = 48; Table 1).^89^

### Glycemic outcomes

The efficacy and safety of GLP-1 RAs in the treatment of T2D have been well-documented over various large trial programs (SUSTAIN,^101^, ^102^, ^103^, ^104^, ^105^, ^106^, ^107^, ^108^, ^109^, ^110^ LEAD,^111^, ^112^, ^113^, ^114^, ^115^, ^116^ SURPASS,^117^, ^118^, ^119^, ^120^, ^121^ AWARD,^96^^,^^122^, ^123^, ^124^, ^125^, ^126^, ^127^, ^128^, ^129^, ^130^, ^131^ DURATION [Diabetes Therapy Utilization: Researching Changes in HbA1c, Weight, and Other Factors Through Intervention with Exenatide Once Weekly]^132^, ^133^, ^134^, ^135^, ^136^, ^137^, ^138^). Across the SUSTAIN trial program, once-weekly subcutaneous semaglutide demonstrated improvements in glycemic control; once-daily subcutaneous liraglutide and once-weekly subcutaneous tirzepatide also demonstrated reductions in glycated hemoglobin across the LEAD^111^, ^112^, ^113^, ^114^, ^115^, ^116^ and SURPASS trials,^117^, ^118^, ^119^, ^120^, ^121^ respectively, all vs placebo or active comparators with various inclusion criteria, demographics, and endpoints. The recent study, GRADE (Glycemia Reduction Approaches in Diabetes: A Comparative Effectiveness Study), published in 2024, investigated the long-term effect of 4 glucose-lowering medications on insulin sensitivity and ß-cell response in people with T2D, with the GLP-1 RA liraglutide demonstrating the greatest effect on ß-cell response after 1 year of treatment compared with insulin glargine, the sulfonylurea glimepiride, and the dipeptidyl peptidase-4 inhibitor sitagliptin (when added to the antihyperglycemic biguanide metformin).^139^

### Weight outcomes

The effects of GLP-1 RAs on weight outcomes in people with overweight and obesity have been extensively investigated. Data from the STEP trial program^140^, ^141^, ^142^, ^143^, ^144^, ^145^ showed that treatment with subcutaneous semaglutide 2.4 mg for overweight and obesity achieved sustained weight loss, together with improvements in CKM syndrome risk factors such as abdominal obesity, hypertension, CKD, and subclinical or clinical CVD,^3^ compared to those treated with placebo.^146^ Treatment with semaglutide was associated with a mean reduction in body weight of 14.9 to 17.4% (STEP 1, 3, 4, and 8) from baseline to week 68 in patients with overweight and/or obesity without T2D.^147^ Similarly, results from the SCALE (Effect of Liraglutide on Body Weight in Non-diabetic Obese Subjects or Overweight Subjects With Co-morbidities: Obesity and Pre-diabetes) program^148^, ^149^, ^150^, ^151^ demonstrated that more than 50% of people with overweight or obesity, with or without T2D, achieved a ≥5% reduction in body weight when treated with liraglutide 3.0 mg vs placebo.^152^ Treatment with tirzepatide 5 mg, 10 mg, and 15 mg demonstrated a mean percentage change in weight of −15.0% (95% CI: −15.9% to −14.2%), −19.5% (95% CI: −20.4% to −18.5%), and −20.9% (95% CI: −21.8% to −19.9%), respectively, vs placebo (*P* < 0.001 for all) in SURMOUNT-1 (Efficacy and Safety of Tirzepatide Once Weekly in Participants Without Type 2 Diabetes Who Have Obesity or Are Overweight With Weight- Related Comorbidities: A Randomized, Double-Blind, Placebo-Controlled Trial).^153^ Furthermore, within all 3 tirzepatide dosage groups, >80% of patients achieved a weight reduction ≥5% vs placebo (35%).^153^ However, in addition to investigations into subcutaneous GLP-1 RAs, oral semaglutide (which has been previously FDA approved as a treatment for T2D)^154^ 3, 7, and 14 mg tablets are FDA-approved to improve glycemic control in patients with T2D as a result of being investigated in the PIONEER program.^77^^,^^155^, ^156^, ^157^, ^158^, ^159^, ^160^, ^161^ Oral semaglutide is currently being investigated in the OASIS program^162^^,^^163^ trial as a treatment in people with T2D and for chronic weight management in people with overweight with at least 1 weight-related complication or obesity. The OASIS 1 trial demonstrated a clinically meaningful decrease in mean body weight with oral semaglutide 50 mg vs placebo (−15.1% vs −2.4%).^162^

### Improvements in heart failure

The effect of once-weekly semaglutide on improvements in patients with HF with preserved ejection fraction (left ventricular ejection fraction ≥45%) and obesity (BMI ≥30 kg/m^2^) vs placebo was investigated in a phase 3 trial (STEP-HFpEF [Effect of Semaglutide 2.4 mg Once Weekly on Function and Symptoms in Subjects With Obesity-related Heart Failure With Preserved Ejection Fraction]); reductions in HF-related symptoms and weight loss were greater in the semaglutide 2.4 mg vs placebo group (mean change in the Kansas City Cardiomyopathy Questionnaire Clinical Summary Score [KCCQ-CSS]). The KCCQ-CSS is a standardized, 23-item, participant-administered instrument that quantifies HF-related symptoms (frequency, severity, and recent changes), physical function, quality of life, and social function. In the phase 3 trial (STEP-HFpEF), mean change in KCCQ-CSS was 16.6 points vs 8.7 points with placebo (estimated difference, 7.8 points; 95% CI: 4.8-10.9; *P* < 0.001) and mean % change in body weight was −13.3% vs −2.6% with placebo (estimated difference −10.7%; 95% CI: −11.9% to −9.4%; *P* < 0.001).^164^ In addition, the effects of once-weekly semaglutide were investigated in patients with HF with preserved ejection fraction (left ventricular ejection fraction ≥45%), obesity (BMI ≥30 kg/m^2^), and T2D.^165^ Treatment with semaglutide led to a greater mean change in the KCCQ-CSS vs placebo (13.7 vs 6.4 points [estimated difference: 7.3 points; 95% CI: 4.1-10.4; *P* < 0.001]) and a greater mean percentage change in body weight vs placebo (−9.8% vs −3.4% [estimated difference: −6.4 percentage points; 95% CI: −7.6 to −5.2; *P* < 0.001]).^166^ The phase 3 SUMMIT (A Randomized, Double-Blind, Placebo-Controlled, Phase 3 Study Comparing the Efficacy and Safety of Tirzepatide Versus Placebo in Patients With Heart Failure With Preserved Ejection Fraction and Obesity) trial^84^ investigated the effect of the once-weekly subcutaneous tirzepatide (up to 15 mg) vs placebo in patients with HF with preserved ejection fraction and obesity; treatment with tirzepatide led to a lower risk of a composite of death from CV causes or worsening HF vs placebo (Table 1). In view of this growing catalog of comprehensive data supporting the use of GLP-1 RAs in the treatment of CKM multimorbidity components, the benefits of several long-acting GLP-1 RAs on various CKM-associated morbidities are currently being investigated further (Table 2).^99^^,^^168^

**Table 2:** Summary of Key Ongoing Trials Investigating Outcomes With GLP-1 RAs in Diseases Within CKM Syndrome

	Population	Treatment Arms	Efficacy Outcomes	Anticipated Study Completion Date
Cardiovascular outcome trials in people with obesity without T2D				
SURMOUNT-MMO^168^	People aged ≥40 years with BMI ≥27 kg/m^2^, established CVD^a^, PAD, or presence of CV risk factors^b^	Escalated doses of tirzepatide 15 mg vs placebo	Primary outcome: • Time to first occurrence of MACE (a composite outcome consisting of all-cause death, nonfatal MI or nonfatal stroke, coronary revascularization or heart failure)	October 2027
Outcome trials in people with MASH				
ESSENCE (part 2)^99^	People aged ≥18 years with biopsy-proven metabolic dysfunction-associated steatohepatitis, liver fibrosis (stage 2 or 3), and nonalcoholic fatty liver disease activity score ≥4	Once-weekly subcutaneous semaglutide 2.4 mg vs placebo	Primary outcome: • Two-part study: time to resolution of steatohepatitis with no worsening of liver fibrosis and reduction in liver fibrosis with no worsening of steatohepatitis (part 1), and cirrhosis-free survival (part 2)	April 2029 (part 2)

For both listed trials in Table 2, in both treatment arms, treatment was in addition to CV risk reduction standard of care for all pertinent comorbid conditions (eg, CVD, T2D) according to local clinical practice guidelines at the time of trial conduct.

Abbreviations as in Table 1.

a Defined as coronary artery or cerebrovascular disease.

b Women aged 55 to 69 years and men aged 50 to 64 years with ≥3 risk factors such as tobacco use, dyslipidemia, or hypertension, or women ≥70 years or men ≥65 years of age with ≥2 risk factors at screening.

## Current recommendations in treatment of disease within the context of CKM syndrome

The FDA has approved 3 GLP-1 RAs for MACE risk reduction in people with T2D. In 2017, liraglutide (0.6-1.8 mg subcutaneously once daily) was approved for the treatment of adults with T2D and established CVD.^170^^,^^171^ In 2020, semaglutide (0.5-1 mg subcutaneously once weekly) was approved for treating adults with T2D and established CVD^172^ with subsequent approval of semaglutide 2.0 mg for treating adults with T2D in 2022.^173^ Dulaglutide (0.75-4.5 mg subcutaneously once weekly) was approved for adults with T2D who have established CVD or multiple CV risk factors.^52^ In 2024, semaglutide 2.4 mg received a FDA label update with an indication for MACE risk reduction in patients with overweight or obesity and established CV disease.^174^ This has subsequently led to the inclusion of GLP-1 RAs in various professional recommendations for the treatment of diseases within the CKM syndrome, including the American Diabetes Association, American College of Cardiology, Kidney Disease Improving Global Outcomes, European Society of Cardiology, American Heart Association, American Association for The Study of Liver Disease, American Gastroenterological Association, and the American Association of Clinical Endocrinology.^3^^,^^175^, ^176^, ^177^, ^178^, ^179^, ^180^, ^181^ However, despite these recommendations and the known benefits of the potential use of GLP-1 RAs in CKM syndrome, GLP-1 RAs remain underutilized within clinical practice, although use has increased in recent years.^182^ This may be due to unfamiliarity with injectable devices or titration methods, concern around patient fear of injections or regarding potential adverse effects, cost/insurance coverage concerns, hesitation regarding dose adjustments required for other glucose-lowering medications or of overstepping interdisciplinary boundaries, and the perception as diabetes medications.^49^^,^^182^^,^^183^ Further considerations impacting uptake of GLP-1 RAs by clinicians include cost and accessibility of treatment. Real-world evidence indicates that people who reside in socioeconomically deprived areas have reduced access to GLP-1 RAs despite these populations having a higher burden of T2D and CVD.^184^ In a 2021 retrospective cohort analysis, low uptake of GLP-1 RAs use was reported among people from racial minority groups and people with low income in the United States, even in people with ASCVD, indicating health care inequalities.^185^ Taking into account cost and accessibility along with the increasingly acknowledged multimodality of the GLP-1 RAs, this class of medication may be a consideration for clinicians in the treatment of patients. A model-based real-world cost-effective analysis of treatment with GLP-1 RAs reported that GLP-1 RAs were deemed a highly cost-effective treatment vs insulin in patients with T2D and prior CVD or CKD in Taiwan.^186^ Within the United States, a cost-effective analysis of 4 GLP-1 RAs (liraglutide 1.8 mg, semaglutide 1.0 mg, dulaglutide 1.5 mg, exenatide 10 μg) for the treatment of obesity found that semaglutide was the most cost-effective, with an incremental cost-effectiveness ratio of $135,467 per quality-adjusted life year and significantly greater weight loss than exenatide, even with the higher outset costs of semaglutide considered.^187^

## Conclusions

Conditions now regarded as CV-kidney-metabolic comorbidities are highly prevalent and seldom occur in isolation, especially given the background influence of overweight and obesity as drivers of chronic disease through metabolic dysregulation. It is important to recognize the inter-relatedness of the morbid manifestations of CKM to encourage clinicians to use a more holistic approach to treating the different components of the syndrome. GLP-1 RAs may be a valuable background therapy to consider in appropriate people where CV-kidney-metabolic disease is diagnosed, given the multisystem benefits of GLP-1 RA treatment. However, GLP-1 RAs remain underused, despite guideline recommendations, due to a variety of factors, including economic and socioeconomic barriers as well as historical treatment patterns. Raising awareness of the multi-organ system benefits of therapeutic classes such as GLP1-RAs for patients with complex comorbidities may lead to greater consideration of these agents for people with CKM syndrome.

## Funding support and author disclosures

Sponsorship for this paper was funded by Novo Nordisk Inc. Dr Lopez-Jimenez is a member of the Scientific Advisory Board at Novo Nordisk Inc and acts in a consulting capacity for Novo Nordisk Inc. Dr Anderson acts as a speaker and in an advisory and consulting capacity for Novo Nordisk Inc, Eli Lilly and Company, Abbott Diabetes Care Inc, 10.13039/100004325AstraZeneca, 10.13039/100001003Boehringer Ingelheim Pharmaceuticals, Inc, Sanofi-Aventis U.S. LLC, and Corcept Therapeutics. Dr Coviello has served as a consultant to Novo Nordisk Inc, Intuitive Surgical Inc, IQVIA, and Wellworks For You Inc. Dr Kumar acts as a speaker and in an advisory and consulting capacity for Novo Nordisk Inc. Dr Bakris acted in a consulting capacity for Novo Nordisk Inc, Alnylam Pharmaceuticals Inc, AstraZeneca Pharmaceuticals LP, and Bayer Corporation.
